# The GLI genes as the molecular switch in disrupting Hedgehog signaling in colon cancer

**DOI:** 10.18632/oncotarget.310

**Published:** 2011-08-21

**Authors:** Tapati Mazumdar, Jennifer DeVecchio, Akwasi Agyeman, Ting Shi, Janet A. Houghton

**Affiliations:** ^1^Department of Cancer Biology, Lerner Research Institute, Cleveland Clinic, Cleveland, OH 44195

**Keywords:** Hedgehog signaling, Colon carcinoma, DNA damage

## Abstract

The Hedgehog (HH) signaling pathway leads to activation of GLI, which transcriptionally regulate target genes. Regulated HH signaling activity is critical during embryogenesis while aberrantly activated HH signaling is evident in a variety of human cancers. Canonical HH signaling engages the transmembrane receptor Patched (PTCH) and the signaling intermediate Smoothened (SMO) to activate GLI1 and GLI2. In addition GLI1 and GLI2 are activated by non-canonical oncogenic signaling pathways to further drive HH-dependent survival. We have demonstrated in human colon carcinoma cells that inhibition of the RAS/RAF pathway by U0126 decreases p-ERK protein expression and also inhibits GLI-luciferase activity and GLI1 mRNA and protein levels. Of importance is the demonstration that targeting of SMO (using cyclopamine) has minimal effect on cell survival in comparison to the inhibition of GLI (using GANT61), which induced extensive cell death in 7/7 human colon carcinoma cell lines. Genetic inhibition of the function of GLI1 and GLI2 by transient transfection of the C-terminus deleted repressor GLI3R, reduced proliferation and induced cleavage of caspase-3 and cell death in HT29 cells, similar to the effects of GANT61. Mechanistically, downstream of GLI1 and GLI2 inhibition, γH2AX (a marker of DNA double strand breaks) expression was upregulated, and γH2AX nuclear foci were demonstrated in cells that expressed GLI3R. Activation of the ATM/Chk2 axis with co-localization of γH2AX and p-Chk2 nuclear foci were demonstrated following GLI1/GLI2 inhibition. GANT61 induced cellular accumulation at G1/S and early S with no further progression before cells became subG1, while cDNA microarray gene profiling demonstrated downregulation of genes involved in DNA replication, the DNA damage response, and DNA repair, mechanisms that are currently being pursued. These studies highlight the importance of targeting the GLI genes downstream of SMO for terminating HH-dependent survival, suggesting that GLI may constitute a molecular switch that determines the balance between cell survival and cell death in human colon carcinoma.

## CANONICAL HEDGEHOG SIGNALING IN CANCER

**Figure 1:**
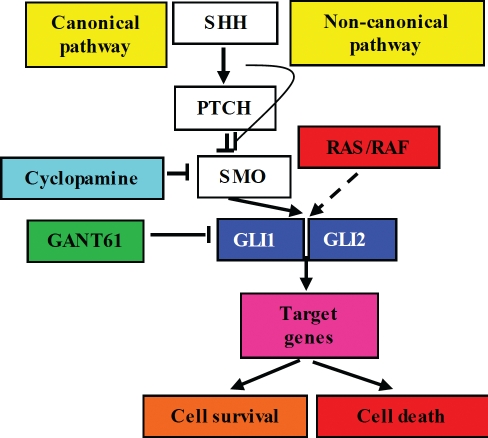
Canonical HH signaling and non-canonical GLI gene activation

Canonical HH signaling engages PTCH, SMO and the GLI family of transcription factors (Figure 1), and in normal cellular processes is involved in embryogenesis, tissue patterning, stem cell function, and differentiation[1, 2]. Several types of human cancers have demonstrated aberrant activation of the HH pathway by ligand-independent signaling such as, amplification of GLI1 or GLI2, mutations in PTCH or SMO, or dysregulated gene expression[1, 3]. In colon cancer, aberrant HH signaling progresses during carcinogenesis and in metastatic disease[4-6], and is also activated in human colon carcinoma cell lines[7-9] and xenograft models[4], by ligand-dependent activation, that occurs in GI cancers[1, 10]. However, the role of HH signaling and its importance in driving cellular survival in colon cancer are not well defined. Small molecule inhibitors of SMO have been studied in preclinical models, and applied to the treatment of various types of cancers in humans[4, 9, 11-14]. Those tumors sensitive to SMO inhibitors, which include basal cell carcinoma[15, 16] and medulloblastoma[11, 17], rely on canonical HH signaling for cellular survival. In other cancer types, SMO inhibitors including GDC-0449, IPI-926 or LDE225, have demonstrated limited clinical activity (reviewed in [11, 12]). Intrinsic resistance to SMO inhibitors is frequent[11-14, 18, 19], and acquired resistance to GDC-0449 following initial response has been reported in medulloblastoma (heterozygous mutation, Asp->His at aa 473 in SMO)[20]. Thus targeting the GLI genes downstream of SMO, that constitute the core of HH-dependent gene regulation, may provide a significant advantage in eliminating HH signaling.

## ACTIVATION OF GLI BY ONCOGENIC, NON-CANONICAL SIGNALING PATHWAYS

**Figure 2:**
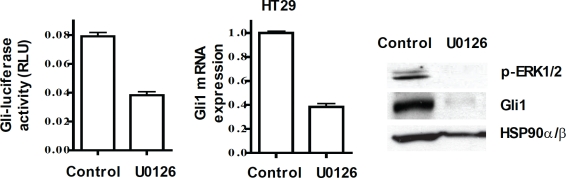
Inhibition of the RAS/RAF pathway by UO126 (20 μM) decreases GLI-luc activity (left), GLI1 mRNA (center), GLI1 and p-ERK1/2 proteins (right)

Non-canonical, oncogene-driven signaling pathways converge on the activation of GLI genes and further converge on their specific downstream targets[3, 18, 21, 22] (see Figure 1). The RAS/RAF/MEK/ERK pathway, with activating mutations in K-RAS or B-RAF that occur in high frequency in colon cancers[23-25], activates GLI function[18, 19, 21]. In HT29 cells (mutated B-RAF V600E[25]), we demonstrated inhibition of GLI-luciferase reporter activity, reduced expression of GLI1 mRNA and protein, and of p-ERK in response to the MEK/ERK and RAS/RAF signaling inhibitor U0126[26, 27] (Figure 2). While loss-of-function mutations in PTCH and gain-of-function mutations in SMO activate HH signaling[1], acquired mutations in SMO or non-canonical GLI activation render cancer cells resistant to SMO antagonists. These observations emphasize the importance of targeting the GLI genes downstream of SMO for terminating HH-dependent survival and inducing cell death in colon carcinoma cells. It therefore follows that termination of HH signaling at the level of GLI may constitute a molecular switch that determines the balance between cell survival or cell death.

## TARGETING GLI1 AND GLI2 WITH GANT61

One of the gaps in our knowledge is that the impact of terminating HH signaling at the level of the GLI genes is unclear. The GLI family of transcription factors regulates target gene expression that determines HH-dependent survival. GLI1 and GLI2 are the primary activators of HH signaling; further, the cooperative roles of GLI1 and GLI2 are critical in the transcriptional regulation of HH target genes[1, 28-30]. While SMO has been extensively investigated as a therapeutic target[11-15, 17], few agents are available that target the GLI genes[31]. GANT61 was identified in a cell-based screen for small molecule inhibitors of GLI1-mediated transcription. In the original study[31], GANT61 1) functions in the nucleus to abrogate GLI function; 2) blocks both GLI1- and GLI2- mediated transcription; 3) in SUFU-null MEFS with constitutively active HH signaling, reduces expression of GLI1 and HIP1 mRNA in contrast to cyclopamine; 4) inhibits GLI1 DNA binding activity (EMSA analysis). We further demonstrated the specificity of GANT61 in targeting GLI1 and GLI2 from reduced GLI1 and GLI2 protein expression[8], inhibition of the binding of GLI1 and GLI2 to the promoter regions of HH target genes, specificity of reduction in GLI-luciferase reporter activity, and rapid inhibition of the transcriptional regulation of the GLI target gene BCL-2, within 1 hr of GANT61 exposure[7].

## INHIBITION OF GLI INDUCES GREATER CYTOTOXICITY THAN TARGETING SMO

**Figure 3:**
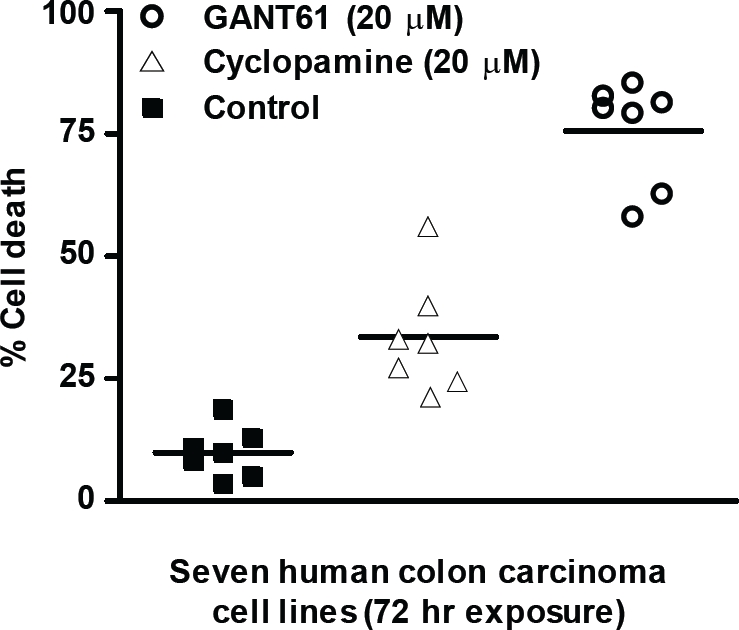
GANT61- and Cyclopamine- induced cell death

GANT61, and the classic SMO inhibitor cyclopamine (for comparison with other model systems), were evaluated in a panel of human colon carcinoma cell lines to determine the impact of inhibiting GLI1/GLI2 vs SMO. Inhibition of GLI1 and GLI2 by GANT61 induced > 80% cell death in 5/7 human colon carcinoma cell lines following 72 hr exposure (20 μM). In contrast, cyclopamine induced < 30% cell death in 6/7 cell lines, at equimolar concentrations (20 μM; Figure 3). In time course studies, 24 hr exposure to GANT61 or 72 hr exposure to cyclopamine was required to initiate cell death[9]. In addition the SMO inhibitor GDC-0449, like cyclopamine, demonstrated limited cytotoxic activity in HT29 cells at equimolar concentrations (unpublished data). Collectively, the data demonstrate increased sensitivity of human colon cancer cells to inhibition of GLI genes compared to that of SMO. This is consistent with the presence of a non-canonical mode of GLI activation in human colon cancer cells. These concentrations and time frames for the induction of cellular effects are similar to those determined in other model systems for inhibitors of HH signaling[4, 19, 33].

## GENETIC DOWNREGULATION OF GLI1 AND GLI2 BY GLI3R PARALLELS CELLULAR EFFECTS MEDIATED BY PHARMACOLOGIC INHIBITION OF GLI1 AND GLI2 BY GANT61

**Figure 4:**
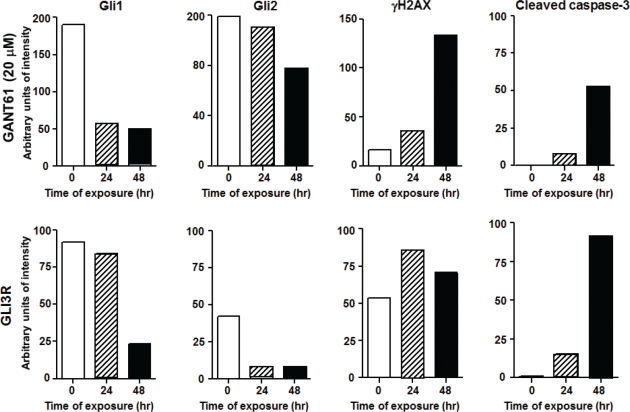
Treatment of HT29 cells with vehicle alone (0.2% DMSO) or GANT61 (20 μM; upper panel), or transient transfection of vector alone, or GLI3R-pCS2-MT[4, 34](lower panel) for 72 hr Expression of proteins was by western analysis and analyzed by densitometry.

A third member of the GLI family is GLI3. A cleaved C-terminally truncated form of GLI3 (GLI3R) demonstrates repressor activity for GLI1 and GLI2 transcriptional regulation, and thereby silences HH-GLI target genes[4, 34]. We confirmed the critical role of GLI1 and GLI2 in cell survival by genetic downregulation of GLI1 and GLI2 following transient transfection of GLI3R[4, 34] in HT29 cells. Transient transfection and expression of GLI3R (GLI3R-pCS2-MT, N-terminal Myc-epitope tag) over a period of 72 hr paralleled the effects of GANT61 by inducing significant changes in cellular morphology, reduced expression of GLI1 and GLI2, reduced proliferation, and induction of cell death. GLI3R-myc expression was detected by 24 hr, with strong expression by 48 hr[32]. Under these conditions of GLI3R transient transfection, GLI1 and GLI2 expression was decreased, with significant cleavage of caspase-3 (Figure 4). To begin to explore the cellular mechanisms downstream of GLI1/GLI2 inhibition that lead to cell death, we demonstrated that γH2AX, a marker of DNA DSBs[35], was expressed in GANT61-treated cells (Figure 4). Further, γH2AX nuclear foci were detected in the cells expressing GLI3R-myc[32]. Thus, both genetic and pharmacologic downregulation of GLI1 and GLI2 induce parallel changes in events leading to cell death, including the induction of γH2AX nuclear foci. One of the critical regulators of DNA damage response is p53, which is stabilized upon DNA damage and modulates multiple components of the DNA damage response pathway[36, 37]. Expression of Gli3R-myc in human colon carcinoma cell lines harboring mutant p53 (functionally inactive) demonstrated DNA damage response suggesting a p53 independent mechanism[[32]]. Further, GANT61 treatment of HT29 cells demonstrated a p21^Cip1^-independent mechanism of cell death[32].

## INHIBITION OF GLI1/GLI2 BY GANT61 INDUCES DNA DAMAGE VIA ATM/CHK2 SIGNALING

**Figure 5:**
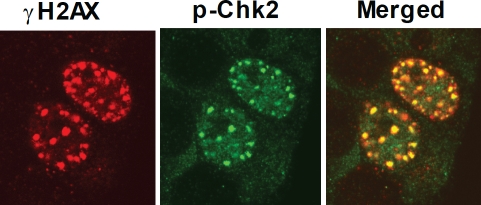
Co-localization of γH2AX and p-Chk2 nuclear foci following GANT61 (20 μM) for 4 hr in HT29 cells

To further investigate the molecular mechanisms underlying DNA damage signaling downstream of GLI1/GLI2 inhibition, HT29 cells treated with GANT61 (20 μM) were examined for expression of the phosphorylated (active) forms of ATM, ATR, Chk1 and Chk2 by Western analysis or by confocal microscopy. p-ATM and p-Chk2 were detected as early as 4 hr, and their expression was sustained for at least 24 hr. In contrast, p-ATR and p-Chk1 expression remained undetectable[32]. p-Chk2 nuclear foci co-localized with γH2AX nuclear foci at the sites of DNA DSBs in GANT61-treated cells (Figure 5). An ATM/Chk2 axis was therefore activated in GANT61-treated human colon carcinoma cells as an early event in the response to DNA damage.

## INHIBITION OF GLI1/GLI2 BY GANT61 INDUCES ARREST IN EARLY S

**Figure 6:**
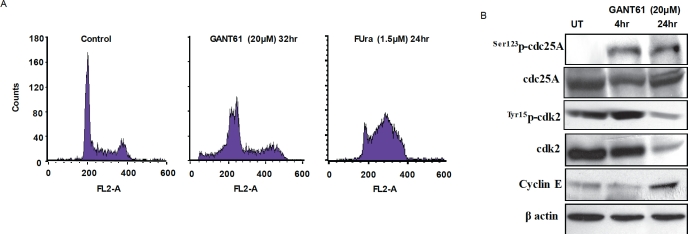
**A**: Flow cytometric analysis of propidium iodide-stained HT29 cells at 0 hr, or after GANT61 (20 μM) for 32 hr[7], or FUra (1.5 μM/leucovorin 1 μM) for 24 hr. **B**: Expression profile of proteins involved in activation of the intra-S-phase checkpoint (western).

Using HT29 cells we have demonstrated that cells treated with GANT61 (20 μM) accumulate in early S-phase and fail to progress further through or beyond early S before becoming subG1[7, 32]. By cell cycle analysis, cells were observed to move from G1 into early S by 32 hr where they remain, detected as two discrete peaks (G1/S, early S; Figure 6A). Analysis by FACS/BrdU incorporation at 32 hr after GANT61 exposure demonstrated a shift in G1/S-phase cells from 8.0% BrdU incorporation in the control to 52.3% at 32 hr, and S-phase cells from 25.0% to 33.6%[32]. In contrast 5-fluorouracil (FUra) combined with leucovorin (1 μM), which targets thymidylate synthase in the inhibition of DNA replication and induction of DNA damage, arrested HT29 cells in mid S-phase[38] (67.0% by FACS/PI staining; Figure 6A), thereby demonstrating the difference in S-phase target location of the mechanism of GANT61-induced inhibition of DNA replication. The cell cycle checkpoints constitute a regulatory mechanism to arrest the cell cycle in response to DNA damage so that cell cycle progression and repair may be temporally coordinated[39-42]. In contrast to the checkpoints at the G1/S and G2/M transitions, the S-phase checkpoint can only delay the progression of S-phase. ATM is the master transducer of the S-phase checkpoint, responds to DNA DSBs, and together with its effector kinase Chk2, is activated in response to GLI1/GLI2 inhibition by GANT61. In activation of the intra-S-phase checkpoint, Cdc25A is phosphorylated on Ser123, which targets this phosphatase for degradation by the proteasome. Activation of Cdk2 and the loading of Cdc45 onto replication origins are inhibited, DNA replication is inhibited, the intra-S-phase checkpoint is activated, and cyclin E accumulates[43-45], as demonstrated in GANT61-treated HT29 cells at 24 hr (Figure 6B). Accumulation of HT29 cells in early S[9](Figure 6A), and the data of Figure 6B, suggest activation of an intra-S-phase checkpoint, which is not sustained.

## GANT61 MODULATES GENES DOWNSTREAM OF GLI1/GLI2 INHIBITION INVOLVED IN THE DNA DAMAGE RESPONSE

To identify unique downstream targets of the GLI genes, analysis with cDNA microarray gene profiling of 18,401 genes identified Differentially Expressed Genes (DEGs) in HT29 (and GC3/c1)[7]. Analyses using GenomeStudio (statistics), Matlab (heat map), Ingenuity (canonical pathway analysis), or by qRT-PCR, identified decreased expression of mRNA for genes involved in DNA replication (including thymidylate synthase, thymidine kinase, topoisomerase2, DNA polymerases, E2F, replication factor C); DNA damage (including H2AX, MDC1, BRCA1, FANCD2, GADD45G, GADD153, REDD1, PCNA); and DNA repair (including RAD51, RAD54, FEN1, MSH6, Exonuclease 1), 24 hr after GANT61 exposure (20 μM; Table 1).

## SUMMARY AND FUTURE DIRECTIONS

We have demonstrated in human colon carcinoma cells that the GLI genes, the transcriptional regulators of the HH signaling response, are activated by both canonical signaling (via SMO) and by non-canonical activation (via the RAS/RAF pathway), which is activated in high frequency in colon cancers. Both pharmacologic and genetic inhibition of GLI function reduced proliferation, GLI1 and GLI2 expression, induced γH2AX expression, cleavage of caspase-3, and cell death. In contrast inhibition of SMO was significantly less effective at inducing cell death in contrast to targeting GLI. DNA damage signaling involved an ATM/Chk2 axis, with Chk2 co-localizing with γH2AX at the sites of DNA DSBs. Further, cells accumulated in early S-phase following GANT61 exposure but did not progress before becoming subG1, suggesting an intra-S-phase checkpoint that could not be sustained. From cDNA microarray gene profiling, genes involved in the inhibition of DNA replication, DNA damage response, and DNA repair were identified downstream of GANT61-induced GLI1/GLI2 inhibition. Chromosome instability is found in the majority of colon cancers, resulting primarily from deregulation of the DNA replication and mitotic spindle checkpoints (reviewed in [45]). Genes involved in canonical HH signaling have been linked to genomic instability, involving inactivation of DNA repair mechanisms, defects in checkpoint activation, and predisposition to development of cancers[46-49]. It therefore follows that termination of HH signaling at the level of GLI may constitute a critical event in determining the balance between cell survival and cell death. The combination of molecular and cellular approaches that are being employed will 1) provide critical new insight into the role of GLI1/GLI2 in driving cellular survival, which has never been addressed in any type of human cancer, and will answer a fundamental question in HH biology, 2) address how dysregulation of the GLI1/GLI2 axis induces DNA damage upstream of cell death or DNA repair upstream of cell survival and 3) provide a framework for the design of strategies and therapeutics specifically targeted at HH signaling via GLI1 and GLI2 in human colon carcinoma.

**Table 1:** Decreased expression of genes downstream of GLI1/GLI2 inhibition following GANT61 (20 μM), 24 hr, HT29 cells; cDNA microarray gene profiling

Function	Fold change	Genes
DNA replication	−2.0 to −4.2	TYMS, TK1, TOP2A, RRM1, RRM2, PRPS2, POLE, POLE2, POLA1, POLA2, POLQ, E2F2, CDT1, PRIM1, GMNN, RFC2, RFC3, RFC4, RFC5
DNA damage response	−1.9 to −4.9	H2AFX, MDC1, BRCA1, FANCD2, BARD1, CDC45L, DDIT2, DDIT3, DDIT4, PPP1R15A, PCNA, ATF3
DNA repair	−1.9 to −4.2	RAD51, RAD51C, RAD54B, RAD54L, FEN1, MSH6, KIAA0101, UNG, LIG1, EXO1, HELLS
